# Supplementary material for: Does the quality of pain relief after major surgery influence the risk of postoperative complications? A prospective observational study
Source: PLoS One. 2025 Sep 23;20(9):e0332866. doi: 10.1371/journal.pone.0332866 (PMC12456833; doi:10.1371/journal.pone.0332866)
Supplement: S1 Table — (DOCX) [file pone.0332866.s001.docx]

**S1 Table**

| **Type of complication** | **ICDs used for implementation (only secondary diagnoses)** |
| --- | --- |
| cardiac | I21.-, I22.-, I46.- |
| pulmonary | J12.-, J13.-, J14.-, J15.-, J16.-, J18.-, J95.1, J95.2; J95.88; J95.9, J96.0, J96.9, U69.01 |
| infectious | A40.-, A41.-, A42.7, A48.3, A49.9, B00.7, B37.6, B37.7, B49.-, R57.2, R65.0, R65.1, T81.4 |
| thormboembolic | I26.-, I63.-, I64.-, I65.-, I66.-, I80.-, I82.2, I82.3, I82.8, I82.9 |
| surgical | T81.0, T81.1, T81.3, T81.7, T81.8, T81.9, T88.8, T88.9, Y84.9 |
