# Supplementary material for: Does the quality of pain relief after major surgery influence the risk of postoperative complications? A prospective observational study
Source: PLoS One. 2025 Sep 23;20(9):e0332866. doi: 10.1371/journal.pone.0332866 (PMC12456833; doi:10.1371/journal.pone.0332866)
Supplement: S2 Table — 27 cases (5%) had to be excluded due to incomplete pain data. Values are number (proportion) or mean (standard deviation) as appropriate. (DOCX) [file pone.0332866.s002.docx]

**S2 Table**

|  | **No pain peaks**  n=411 | **Pain peaks NRS >6**  n=101 | **P-value** |
| --- | --- | --- | --- |
| **Sex**  male | 153 (33%) | 34 (34%) | 0.505 |
| **Mean age**; y | 66 (14) | 65 (15) | 0.911 |
| **ASA physical status**  1  2  3  4 | 25 (6%)  215 (52%)  163 (40%)  8 (2%) | 1 (1%)  54 (54%)  43 (43%)  3 (3%) | 0.135 |
| **Mean BMI**; kg.m^-1^ | 28 (6) | 29 (6) | 0.257 |
| **Chronic pain syndrome** | 32 (8%) | 13 (13%) | 0.106 |
| **Pain medication within 6 months prior to surgery**  opioids  non opioids  co-analgesics | 59 (14%)  157 (38%)  30 (7%) | 29 (29%)  57 (56%)  13 (13%) | 0.001  0.001  0.071 |
| **Type of surgery**  endoprosthetic  bone  major general  thoracic  laparoscopic  others | 129 (31%  84 (20%)  108 (26%)  23 (6%)  24 (6%)  43 (11%) | 57 (56%)  22 (22%)  11 (11%)  3 (3%)  5 (5%)  3 (3%) | <0.001 |
| **Acute pain treatment**  patient controlled intravenous  regional anesthesia, continuous  regional anesthesia, single shot  others | 36 (9%)  221 (54%)  147 (34%)  7 (2%) | 10 (10%)  35 (35%)  55 (55%)  1 (1%) | 0.003 |
| **Mean time to sustained adequate pain relief**; h | 68 (51) | 79 (44) | <0.001 |
