# Supplementary material for: Does the quality of pain relief after major surgery influence the risk of postoperative complications? A prospective observational study
Source: PLoS One. 2025 Sep 23;20(9):e0332866. doi: 10.1371/journal.pone.0332866 (PMC12456833; doi:10.1371/journal.pone.0332866)
Supplement: S3 Table — Values are numbers and proportions. (DOCX) [file pone.0332866.s003.docx]

**S3 Table**

|  | **No pain peaks**  n=413 | **Pain peaks NRS >6**  n=101 | **P-value** |
| --- | --- | --- | --- |
| **Inpatient complications**  cardiac  pulmonary  infectious  thromboembolic  surgical  Composite: at least one of the above complications | 2 (1%)  49 (12%)  14 (3%)  12 (3%)  29 (7%)  75 (18%) | 0 (0%)  6 (6%)  3 (3%)  1 (1%)  9 (9%)  16 (16%) | 1.000  0.082  1.000  0.480  0.524  0.571 |
| **Postoperative use of analgesics for at least 6 months**  opioids  non opioids  co-analgesics  Composite: any of the above | 18 (4%)  55 (14%)  11 (3%)  67 (16%) | 10 (10%)  16 (16%)  7 (7%)  25 (25%) | 0.029  0.522  0.063  **0.048** |
