# Supplementary material for: Does the quality of pain relief after major surgery influence the risk of postoperative complications? A prospective observational study
Source: PLoS One. 2025 Sep 23;20(9):e0332866. doi: 10.1371/journal.pone.0332866 (PMC12456833; doi:10.1371/journal.pone.0332866)
Supplement: S4 Table — Rapid pain recovery was defined as a time to reach sustained pain scores below 4 (NRS) with movement below the median time of the group. Times above the median were classified as slow pain recovery. Values are number (proportion) or mean (standard deviation) as appropriate. (DOCX) [file pone.0332866.s004.docx]

**S4 Table**

|  | **Fast pain recovery**  n=337 | **Slow pain recovery**  n=202 | **P-value** |
| --- | --- | --- | --- |
| **Sex**  male | 119 (35%) | 79 (39%) | 0.376 |
| **Mean age**; y | 65 (15) | 67 (14) | 0.344 |
| **ASA physical status**  1  2  3  4 | 21 (6%)  182 (54%)  125 (37%)  9 (3%) | 6 (3%)  99 (49%)  95 (47%)  2 (1%) | 0.048 |
| **Mean BMI**; kg.m^-1^ | 29 (6) | 28 (6) | 0.025 |
| **Chronic pain syndrome** | 26 (8%) | 21 (10%) | 0.286 |
| **Pain medication within 6 months prior to surgery**  opioids  non opioids  co-analgesics | 50 (15%)  133 (40%)  26 (8% | 40 (20%)  91 (45%)  18 (9%) | 0.135  0.203  0.624 |
| **Type of surgery**  endoprosthetic  bone  major general  thoracic  laparoscopic  others | 112 (33%)  88 (26%)  62 (18%)  8 (2%)  22 (7%)  45 (13%) | 77 (38%)  29 (14%)  65 (32%)  18 (9%)  10 (5%)  3 (2%) | <0.001 |
| **Acute pain treatment**  patient controlled intravenous  regional anesthesia, continuous  regional anesthesia, single shot  others | 33 (10%)  152 (45%)  142 (42%)  10 (3%) | 19 (9%)  116 (57%)  67 (33%)  0 (0%) | 0.004 |
| **Mean time to sustained adequate pain relief**; h | 44 (18) | 113 (59) | <0.001 |
| **Presence of pain peaks NRS >6 during postoperative days 1-3**  *Definition not applicable due to incomplete pain data* | 49 (16%)  *20* | 52 (27%)  *7* | 0.002 |
