# Supplementary material for: Does the quality of pain relief after major surgery influence the risk of postoperative complications? A prospective observational study
Source: PLoS One. 2025 Sep 23;20(9):e0332866. doi: 10.1371/journal.pone.0332866 (PMC12456833; doi:10.1371/journal.pone.0332866)
Supplement: S5 Table — Rapid pain recovery was defined as a time to reach sustained pain scores below 4 (NRS) with movement below the median time of the group. Times above the median were classified as slow pain recovery. Values are numbers and proportions. (DOCX) [file pone.0332866.s005.docx]

**S5 Table**

|  | **Fast pain recovery**  n=337 | **Slow pain recovery**  n=202 | **P-value** |
| --- | --- | --- | --- |
| **Inpatient complications**  cardiac  pulmonary  infectious  thromboembolic  surgical  Composite: at least one of the above complications | 2 (1%)  29 (9%)  8 (2%)  7 (2%)  15 (5%)  45 (13%) | 0 (0%)  32 (16%)  13 (6%)  8 (4%)  26 (13%)  55 (27%) | 0.531  0.010  0.018  0.198  0.0004  0.0001 |
| **Postoperative use of analgesics for at least 6 months**  opioids  non opioids  co-analgesics  Composite: any of the above | 17 (5%)  45 (13%)  11 (3%)  56 (17%) | 11 (5%)  27 (13%)  7 (4%)  37 (18%) | 0.839  0.997  0.900  0.613 |
