# Supplementary material for: Does the quality of pain relief after major surgery influence the risk of postoperative complications? A prospective observational study
Source: PLoS One. 2025 Sep 23;20(9):e0332866. doi: 10.1371/journal.pone.0332866 (PMC12456833; doi:10.1371/journal.pone.0332866)
Supplement: S6 Table — Inadequately controlled pain was defined as NRS values with movement ≥4 at least once within postoperative days 1–3, pain peaks as NRS values >6 with movement at least once within the first three postoperative days, and slow pain recovery as a time to reach sustained pain scores below 4 (NRS) with movement above the median time of the study population. aAdjusted for sex, age, BMI, ASA score, and type of surgery. bAdjusted for sex, age, BMI, ASA score, diagnosis of chronic pain syndrome at the time of hospital admission, opioid medication within 6 months before surgery, nonopioid medication within 6 months before surgery, co-analgesics medication within 6 months before surgery, and type of surgery. (DOCX) [file pone.0332866.s006.docx]

**S6 Table**

|  | **Inadequately controlled pain**  **NRS >3**  n=376 | **P-value** | **pain peaks**  **NRS >6**  n=101 | **P-value** | **Slow pain recovery**  n=202 | **P-value** |
| --- | --- | --- | --- | --- | --- | --- |
| **Inpatient complications**  Crude OR (95% CI)  Adjusted^a^ OR (95% CI)  AME^a^ (95% CI)  **Prolonged postoperative use of analgesics**  Crude OR (95% CI)  Adjusted^b^ OR (95% CI)  AME^b^ (95% CI) | 1.88 (1.10-3.35)  2.56 (1.43-4.80)  0.12 (0.05-0.20)  2.11 (1.21-3.87)  1.87 (0.98-3.72)  0.07 (-0.004-0.15) | <0.001  0.002  0.002  0.011  0.064  0.062 | 0.84 (0.45-1.49)  1.27 (0.64-2.42)  0.03 (-0.05-0.12)  1.69 (0.99-2.82)  1.20 (0.65-2.19)  0.02 (-0.05-0.10) | 0.571  0.478  0.478  0.049  0.555  0.555 | 2.43 (1.56-3.79)  2.21 (1.35-3.64)  0.10 (0.04-0.16)  1.13 (0.71-1.77)  0.95 (0.55-1.60)  -0.01 (-0.07-0.06) | <0.001  0.002  0.001  0.613  0.836  0.836 |
