# Supplementary material for: Does the quality of pain relief after major surgery influence the risk of postoperative complications? A prospective observational study
Source: PLoS One. 2025 Sep 23;20(9):e0332866. doi: 10.1371/journal.pone.0332866 (PMC12456833; doi:10.1371/journal.pone.0332866)
Supplement: S7 Tables — (DOCX) [file pone.0332866.s007.docx]

| **a)** Inadequately controlled postoperative pain and postoperative complications | | | |
| --- | --- | --- | --- |
|  | **Composite postoperative complications** | | |
| *Predictors* | *Odds Ratio* | *95% CI* | *p* |
| (Intercept) | 0.00 | 0.00 – 0.04 | **<0.001** |
| **Inadequately controlled postoperative**  **pain** | 2.56 | 1.43 – 4.80 | **0.002** |
| Age | 1.03 | 1.01 – 1.05 | **0.006** |
| Sex, female | 0.63 | 0.39 – 1.02 | 0.058 |
| BMI | 1.03 | 0.99 – 1.07 | 0.155 |
| ASA 1 | - | - | - |
| ASA 2 | 0.74 | 0.22 – 3.41 | 0.656 |
| ASA 3 | 1.04 | 0.29 – 4.98 | 0.954 |
| ASA 4 | 1.49 | 0.22 – 10.48 | 0.678 |
| Surgery type: endoprosthetic | - | - | - |
| Surgery type: bone | 2.75 | 1.29– 5.88 | **0.009** |
| Surgery type: major abdominal | 8.12 | 4.14 – 16.52 | **<0.001** |
| Surgery type: thoracic | 2.58 | 0.75 – 7.81 | 0.108 |
| Surgery type: others | 1.12 | 0.25 – 3.73 | 0.866 |
| Surgery type: laparoscopic | 4.18 | 1.41 – 11.55 | **0.007** |
| Observations | 523 | | |
| R^2^ Tjur | 0.135 | | |

| **b)** Postoperative pain peaks and postoperative complications | | | |
| --- | --- | --- | --- |
|  | **Composite postoperative complications** | | |
| *Predictors* | *Odds Ratio* | *95% CI* | *p* |
| (Intercept) | 0.01 | 0.00 – 0.12 | **<0.001** |
| **Postoperative pain peaks** | 1.27 | 0.64 – 2.42 | 0.478 |
| Age | 1.03 | 1.01 – 1.05 | **0.005** |
| Sex, female | 0.61 | 0.38 – 1.00 | 0.050 |
| BMI | 1.02 | 0.98 – 1.07 | 0.332 |
| ASA 1 | - | - | - |
| ASA 2 | 0.62 | 0.18 – 2.87 | 0.481 |
| ASA 3 | 0.84 | 0.23 – 4.06 | 0.806 |
| ASA 4 | 1.23 | 0.17 – 8.79 | 0.831 |
| Surgery type: endoprosthetic | - | - | - |
| Surgery type: bone | 2.16 | 1.00 – 4.68 | **0.049** |
| Surgery type: major abdominal | 6.86 | 3.44 – 14.22 | **<0.001** |
| Surgery type: thoracic | 2.61 | 0.75 – 7.98 | 0.106 |
| Surgery type: others | 0.83 | 0.18 – 2.68 | 0.772 |
| Surgery type: laparoscopic | 3.17 | 1.02 – 8.96 | **0.035** |
| Observations | 512 | | |
| R^2^ Tjur | 0.112 | | |

| c) Slow postoperative pain recovery and postoperative complications | | | |
| --- | --- | --- | --- |
|  | **Composite postoperative complications** | | |
| *Predictors* | *Odds Ratio* | *95% CI* | *p* |
| (Intercept) | 0.00 | 0.00 – 0.05 | **<0.001** |
| **Slow postoperative pain recovery** | 2.21 | 1.35 – 3.64 | **0.002** |
| Age | 1.03 | 1.01 – 1.05 | **0.004** |
| Sex, female | 0.67 | 0.41 – 1.08 | 0.095 |
| BMI | 1.04 | 1.00 – 1.08 | 0.064 |
| ASA 1 | - | - | - |
| ASA 2 | 0.59 | 0.17 – 2.72 | 0.433 |
| ASA 3 | 0.76 | 0.21 – 3.65 | 0.696 |
| ASA 4 | 1.34 | 0.19 – 9.55 | 0.765 |
| Surgery type: endoprosthetic | - | - | - |
| Surgery type: bone | 2.67 | 1.25 – 5.73 | **0.011** |
| Surgery type: major abdominal | 7.58 | 3.93 – 15.19 | **<0.001** |
| Surgery type: thoracic | 2.20 | 0.64 – 6.71 | 0.181 |
| Surgery type: others | 1.04 | 0.23 – 3.41 | 0.954 |
| Surgery type: laparoscopic | 3.73 | 1.27 – 10.21 | **0.012** |
| Observations | 539 | | |
| R^2^ Tjur | 0.146 | | |

| d) Inadequately controlled postoperative pain and prolonged postoperative analgesic use | | | |
| --- | --- | --- | --- |
|  | **prolonged postoperative analgesic use** | | |
| *Predictors* | *Odds Ratio* | *95% CI* | *p* |
| (Intercept) | 0.02 | 0.00 – 0.48 | **0.022** |
| **Inadequately controlled postoperative pain** | 1.87 | 0.98 – 3.72 | 0.064 |
| Age | 1.02 | 0.99 – 1.04 | 0.157 |
| Sex, female | 1.15 | 0.68 – 1.99 | 0.602 |
| BMI | 1.03 | 0.98 – 1.07 | 0.211 |
| ASA 1 | - | - | - |
| ASA 2 | 4.23 | 0.77 – 79.55 | 0.178 |
| ASA 3 | 4.19 | 0.72 – 80.61 | 0.190 |
| ASA 4 | 1.14 | 0.04 – 34.45 | 0.932 |
| Chronic pain syndrome | 2.11 | 0.99 – 4.40 | **0.048** |
| No preoperative opioid use | 0.68 | 0.38 – 1.25 | 0.205 |
| No preoperative nonopioid use | 0.26 | 0.15 – 0.45 | **<0.001** |
| No preoperative co-analgesics use | 0.33 | 0.16 – 0.71 | **0.004** |
| Surgery type: endoprosthetic | - | - | **-** |
| Surgery type: bone | 1.81 | 0.88 – 3.73 | 0.106 |
| Surgery type: major abdominal | 1.74 | 0.86 – 3.54 | 0.124 |
| Surgery type: thoracic | 0.58 | 0.08 – 2.45 | 0.511 |
| Surgery type: others | 1.19 | 0.39 – 3.30 | 0.743 |
| Surgery type: laparoscopic | 0.00 | 0.00 – 0.00 | 0.982 |
| Observations | 523 | | |
| R^2^ Tjur | 0.178 | | |

| **e)** Postoperative pain peaks and prolonged postoperative analgesic use | | | |
| --- | --- | --- | --- |
|  | **prolonged postoperative analgesic use** | | |
| *Predictors* | *Odds Ratio* | *95% CI* | *p* |
| (Intercept) | 0.06 | 0.00 – 1.00 | 0.068 |
| **Postoperative pain peaks** | 1.20 | 0.65 – 2.19 | 0.555 |
| Age | 1.01 | 0.99 – 1.04 | 0.248 |
| Sex, female | 1.18 | 0.69 – 2.03 | 0.553 |
| BMI | 1.03 | 0.98 – 1.07 | 0.230 |
| ASA 1 | - | - | - |
| ASA 2 | 3.91 | 0.71 – 73.67 | 0.203 |
| ASA 3 | 3.68 | 0.62 – 71.10 | 0.236 |
| ASA 4 | 0.93 | 0.03 – 29.38 | 0.965 |
| Chronic pain syndrome | 2.03 | 0.96 – 4.20 | 0.060 |
| No preoperative opioid use | 0.64 | 0.36 – 1.18 | 0.150 |
| No preoperative nonopioid use | 0.27 | 0.15 – 0.46 | **<0.001** |
| No preoperative co-analgesics use | 0.32 | 0.15 – 0.68 | **0.003** |
| Surgery type: endoprosthetic | - | - | **-** |
| Surgery type: bone | 1.67 | 0.81 – 3.41 | 0.160 |
| Surgery type: major abdominal | 1.58 | 0.76 – 2.26 | 0.215 |
| Surgery type: thoracic | 0.57 | 0.08 – 2.50 | 0.506 |
| Surgery type: others | 0.98 | 0.32 – 2.61 | 0.962 |
| Surgery type: laparoscopic | 0.00 | 0.00 – 5344.66 | 0.983 |
| Observations | 512 | | |
| R^2^ Tjur | 0.172 | | |

| **f)** Slow postoperative pain recovery and prolonged postoperative analgesic use | | | |
| --- | --- | --- | --- |
|  | **prolonged postoperative analgesic use** | | |
| *Predictors* | *Odds Ratio* | *95% CI* | *p* |
| (Intercept) | 0.07 | 0.00 – 1.20 | 0.088 |
| **Slow postoperative pain recovery** | 0.95 | 0.55 – 1.60 | 0.836 |
| Age | 1.01 | 0.99 – 1.03 | 0.268 |
| Sex, female | 1.11 | 0.66 – 1.90 | 0.692 |
| BMI | 1.03 | 0.98 – 1.07 | 0.203 |
| ASA 1 | - | - | - |
| ASA 2 | 3.76 | 0.69 – 70.32 | 0.215 |
| ASA 3 | 3.65 | 0.63 – 69.96 | 0.234 |
| ASA 4 | 0.93 | 0.03 – 29.72 | 0.966 |
| Chronic pain syndrome | 2.03 | 0.97 – 4.16 | 0.057 |
| No preoperative opioid use | 0.63 | 0.35 – 1.14 | 0.120 |
| No preoperative nonopioid use | 0.26 | 0.15 – 0.45 | **<0.001** |
| No preoperative co-analgesics use | 0.31 | 0.15 – 0.67 | **0.002** |
| Surgery type: endoprosthetic | - | - | **-** |
| Surgery type: bone | 1.40 | 0.69 – 2.82 | 0.350 |
| Surgery type: major abdominal | 1.46 | 0.73 – 2.91 | 0.275 |
| Surgery type: thoracic | 0.55 | 0.08 – 2.40 | 0.480 |
| Surgery type: others | 0.86 | 0.28 – 2.29 | 0.776 |
| Surgery type: laparoscopic | 0.00 | 0.00 – 3099.43 | 0.982 |
| Observations | 539 | | |
| R^2^ Tjur | 0.168 | | |
