# Supplementary material for: Does the quality of pain relief after major surgery influence the risk of postoperative complications? A prospective observational study
Source: PLoS One. 2025 Sep 23;20(9):e0332866. doi: 10.1371/journal.pone.0332866 (PMC12456833; doi:10.1371/journal.pone.0332866)
Supplement: Attachment — Submitted filename: Review.docx [file pone.0332866.s008.docx]

Review PONE-D-25-25651

Quality of pain release after major surgery and its association with postoperative complications – a prospective observational study

In general:

In this prospective multicenter observational study the authors tested the hypothesis that postoperative complications occur more frequently in patients with poor pain control regarding both severity and duration. Second, that the likelihood of pain chronification is higher in these patients, reflected by prolonged postoperative analgesic consumption.

The study is well-designed and makes original use of various linked registries. Considering the individual time that a patient needs to overcome postoperative pain, which means having stable, at most mild pain levels, as a new approach is interesting.

In the use of the registries, understandable methodological choices have been made, which may, however, influence the results. I therefore suggest examining these choices carefully and providing a more detailed discussion of potential limitations and points for consideration."

Major

Methods:

The inclusion of only patients insured with BARMER GEK may introduce a risk of selection bias, as membership in this statutory health insurance fund may not be representative of the general German population. Individuals insured by BARMER may differ systematically from those covered by other statutory or private insurers with respect to socioeconomic status, age, health status, or regional distribution. For example, higher-income individuals are more likely to opt for private health insurance, and BARMER may have a higher proportion of older or chronically ill patients compared to smaller or more specialized funds. Therefore, the study findings may have limited generalizability beyond the insured population of BARMER GEK and introducing other risk factors for postoperative pain. I suggest to comment this in the discussion section.

When considering patients to have inadequately controlled postoperative pain if the NRS scores with movement were above three at least once on days 1-3 after surgery (according to the cut-off for moderate to severe pain described by Gerbershagen), this means that patients with an NRS4 once in those three days are considered to have inadequate pain control. Although used in other studies, to my clinical opinion this is far to strict and may have lead to more patients in the inadequate pain control group. When checking literature, the results of the paper of van Boekel et al.(2017) reinforces my clinical opinion. I would suggest not only stating that the cut-off is widely used, but also discussing the cut-off in the light of personalized levels of pain scores.

van Boekel, R. L. M., Vissers, K. C. P., van der Sande, R., Bronkhorst, E., Lerou, J. G. C., & Steegers, M. A. H. (2017). Moving beyond pain scores: Multidimensional pain assessment is essential for adequate pain management after surgery. PloS one, 12(5), e0177345. https://doi.org/10.1371/journal.pone.0177345

Results:

Mentioned in text of results (page 15, line 265): “In patients with inadequate pain control, at least one postoperative complication occurred more frequently (22%) than in the adequately treated group (16%).” (p-value missing). However, in table 2 it shows that “Composite: at least one of the above complications” is 21% versus 12%? Please explain.

Mentioned in text of results (page 18, line 316): “Pain peaks during the first three postoperative days and the speed of pain recovery had no influence on the occurrence of chronic postoperative pain in our analysis (pain peaks: 0.02; 95% CI -0.05 to 0.10; P=0.555; pain recovery: -0.01; 95% 319 CI -0.07 to 0.06; P=0.836).” I think you mean “prolonged postoperative analgesic use“ as a proxy for chronic postoperative pain, as stated in methods? Please explain.

Discussion:

You were unable to demonstrate any influence of the aforementioned pain therapy characteristics on prolonged postoperative analgesic use. I would suggest to add a paragraph in the discussion to discuss prolonged postoperative analgesic use as a proxy for chronic postoperative pain, in the light oft he German culture of opioid prescribing which may differ from other countries?

Minor:

Abstract:

Please correct objektives in objectives

Please add % to numbers of patients in results, for example page 16, line 270. And p-values when comparing results, for example page 15, line 261.

In results is steated: “were more than twice as high for patients with inadequately controlled pain (adjOR 2.56; 95% CI 1.43 266 to 4.80, P=0.002).” The same results are described in the abstract as: “had almost three times the risk of complications (adjOR 2.56; 48 95% CI 1.43-4.80, p=0.002)”. In my opinion, 2.56 is not almost 3, please adjust.

Table 1, what do you mean with “Definition not applicable” underneath table 1?
