# Supplementary material for: Does the quality of pain relief after major surgery influence the risk of postoperative complications? A prospective observational study
Source: PLoS One. 2025 Sep 23;20(9):e0332866. doi: 10.1371/journal.pone.0332866 (PMC12456833; doi:10.1371/journal.pone.0332866)
Supplement: Attachment — Submitted filename: Response to Reviewers.docx [file pone.0332866.s009.docx]

Many thanks to the academic editor, Dr. Alexander Wolf, for the opportunity to revise our manuscript. We have now complied with the journal's style requirements and added the necessary information about the funder and data availability.

We would also like to thank the two reviewers for taking the time to read our work carefully and for their very helpful and appropriate comments. Below are our responses to the individual points that were addressed.

Response to Reviewers

Reviewer #1:

Dear authors, this is a well-conducted prospective observational study evaluating whether poor postoperative pain control is associated with higher rates of complications and prolonged analgesic use. Very nice initiative while research on this topic remains challenging.
In the age of ever-increasing knowledge regarding perioperative safety, I appreciate the opportunity to carefully read and comment regarding the submission “Quality of pain release after major surgery and its association with postoperative complications – a prospective observational study”. I really like the idea of studying. I congratulate the authors for the large amount of analyzed data. Overall, the study is well reported and complete.

> Thank you very much for this positive assessment.

Major comments:

1.1. Although the study is observational, the language in some parts (e.g., “postoperative pain causes complications”) could be misinterpreted. Clarify that findings show associations, not causation. From my early view, I feel the aim (and ambition) here is an explanation and causal inference. Let us say, ¿is poorly controlled postop pain causing postop. complications? However, during my lecture of the manuscript I have seen only a few details about causal interpretation of the calculated estimates with some attempts to avoid the word “cause.” Moreover, the analytic intentions were focused on association which is fine but the interpretation -in some aspects- is causal and that should be clarified. I consider the interpretation of the estimates (and confidence intervals) could be improved -and there are plenty of recent literature to help this way- (i.e. doi:10.2105/AJPH.2018.304337). Are you sure you just want to say, “associated with”. Following this line, the main abstract conclusion is causal.

> We thank the reviewer for this comment, as it highlights an essential issue in medical, behavioral, and social sciences. Since both this comment and the subsequent ones address the topic of causality, we would like to offer a general statement at this point. First, our idea is in fact that some of the stochastic relationships we anticipated in our data arise from underlying causal relationships. For instance, we believe that effective postoperative pain management can significantly influence the risk of various short- and long-term consequences. Second, although it is not explicitly stated, our statistical reasoning is based on the Potential Outcome Framework, also called the Neyman-Rubin Causal Model (NRCM) [1, 2]. According to the NRCM, confounding variables are pre-treatment factors that (a) influence the assignment to the treatment group and (b) also affect the outcome variable. In perfectly randomized studies, confounding variables can be eliminated through the study design, as the mechanism for allocation is stochastically independent of all pretreatment variables and potential outcomes. In this scenario, both unconditional and conditional group comparisons (e.g., mean differences) serve as unbiased estimates of the average and conditional average treatment effects.

In our study, adequate postoperative pain control (as opposed to inadequate pain control) is the exposure variable that relates to the treatment variable in the NRCM. Random assignment to a pain therapy group and a control group in a clinical trial is possible, but random assignment to the “adequacy/inadequacy” of a pain control is not. The presence of confounding variables, as defined in the NRCM, cannot therefore be ruled out. This means that the data resulting from our study design allow for statistical analysis of stochastic relationships, but not per se of causal inference. The NRCM outlines the conditions or assumptions necessary to establish causal conclusions in non-randomized or observational studies, which are for example the Stable Unit Treatment Value Assumption, strong ignorability (i.e., conditional exchangeability), and common support/overlap/positivity.

The assumption of strong ignorability is essential to our argument. This assumption states that the assignment mechanism is conditionally independent of the actual outcome variables when accounting for confounding variables. In simpler terms, strong ignorability means that the assignment of individuals to treatment groups (e.g. “adequacy/inadequacy” of postoperative pain control in our analysis) is random across all combinations of covariates, similar to a randomized experiment. Some researchers refer to this assumption as conditional exchangeability [3].

Unfortunately, strong ignorability is not an assumption that can be tested empirically. A conscientious researcher can only select potential confounding variables as comprehensively and theoretically sound as possible and incorporate them appropriately into the statistical analysis. However, this does not eliminate the possibility of residual bias, which is the systematic difference between the effect estimator and the true causal effect. This bias also cannot be quantified empirically. This potential and unknown bias seems to be precisely the reason why, in our experience, causal formulations are undesirable, if not a taboo, in observational studies. The toning down or even avoidance of causal statements in non-randomized studies is often used to avoid immediate rejection of manuscripts. This is a well-known and, fortunately, now controversially discussed topic.

The reviewer kindly gave a reference (Hernan, 2018; doi:10.2105/AJPH.2018.304337). Another example is Grosz et al. [4]:

“*Causal inference is a central goal of research. However, most psychologists refrain from explicitly addressing causal research questions and avoid drawing causal inference on the basis of nonexperimental evidence. We argue that this taboo against causal inference in nonexperimental psychology impairs study design and data analysis, holds back cumulative research, leads to a disconnect between original findings and how they are interpreted in subsequent work, […]*”.

This statement applies equally to medical research. In light of the reviewer's comments and the cited literature, we feel encouraged to clearly formulate our hypotheses as causal relationships. Of course, we also discuss the limitations regarding causal conclusions based on the study design. We have therefore fundamentally revised the manuscript:

First, we adjusted the title of the manuscript (*Does the quality of pain relief after major surgery influence the risk of postoperative complications? A prospective observational study*).

Second, we carefully reviewed the manuscript for consistency in wording (causality/correlation). In the revised version, we clearly stated that we (a) assume an influence of (in)adequate pain management on the risk of postoperative complications, (b) took into account a set of potentially confounding variables due to the non-randomized design, and (c) discuss the limitations regarding causal conclusions in the discussion section of the manuscript.

Subsequently, we have revised the last paragraph in the introduction section:

*“We therefore hypothesize that adequate pain control in hospitalized patients undergoing major surgery influences the risk of postoperative complications. Accordingly, we tested the following hypotheses: (a) postoperative complications occur more frequently in patients with poor pain control regarding both severity and duration, (b) the risk of pain chronification, reflected in prolonged postoperative analgesic use, is higher in patients with poor pain control.”* Please note that we explicitly mention the target population here.

We have also added the following paragraph to the discussion:

*“We suspect that adequate pain control after major surgery has an influence on the risk of postoperative complications. However, drawing causal conclusions from observational studies relies on certain assumptions, as outlined in the Neyman-Rubin causal model (NRCM). The Average Marginal Effect (AME), derived from the logistic regression model, serves as an unbiased estimator of the Average Treatment Effect (ATE) under the conditions that (a) all confounding variables are properly accounted for (i.e., strong ignorability and correct model specification) and that these covariates are accurately measured. However, due to the study's design, we cannot completely eliminate the possibility of unmeasured confounders, which can introduce residual bias in the adjusted odds ratio (OR) and AME”.*

Finally, it is important to note that the word “association” or expressions such as “associated with” have not been completely removed. We have intentionally avoided using causal language, particularly when referencing the findings of other studies, as this may not reflect the original authors' intentions. Consequently, when we refer to an “association”, we do not dismiss the possibility of a causal effect, especially since a causal relationship implies (at least) a stochastic (sometimes even deterministic) dependence between the outcome and the cause.

[1] Rubin, D. B. (1974). Estimating causal effects of treatments in randomized and nonrandomized studies. Journal of Educational Psychology, 66, 688-701. <https://doi.org/doi.org/10.1037/h0037350>

[2] Holland, P. W. (1986). Statistics and Causal Inference. Journal of the American statistical association, 81(396), 945-960. <https://doi.org/10.1080/01621459.1986.10478354>

[3] Hernán, M. A., & Robins, J. M. (2006). Estimating causal effects from epidemiological data. Journal of Epidemiology and Community Health, 60(7), 578-586. <https://doi.org/10.1136/jech.2004.029496>

[4] Grosz, M. P., Rohrer, J. M., & Thoemmes, F. (2020). The Taboo Against Explicit Causal Inference in Nonexperimental Psychology. Perspectives on Psychological Science, 15(5), 1243-1255. <https://doi.org/10.1177/1745691620921521>

1.2.	I suggest you should state why your intentions are not directly causal (if it is what you were aiming for). Target trial strategy where a causal analysis in observational data can be viewed as an attempt to emulate a hypothetical trial—the target trial could be useful (doi: 10.1093/aje/kwv254).

> As explained in the previous response, we were very cautious in our use of causal formulations. In the revised version, we clarify our assumption regarding the causal relationship between (in)adequate pain control after major surgery and postoperative complications. We believe that target trial emulation (TTE) is a promising and effective method for investigating the effectiveness of treatments (i.e. causal effects) in observational studies. Additionally, it can help explicitly formulate causal research questions in such studies. However, TTE requires a level of scientific rigor equivalent to that of a randomized controlled trial (RCT), which we were unable to fully achieve in our study based on primary data. We also question whether the “appropriateness” of a therapy can be considered a treatment variable in the context of the existing literature on TTE [1,2]. Moreover, we feel that it would be misleading to retrospectively characterize our study as an emulated target trial. Nevertheless, our study does incorporate elements of TTE. Most importantly, we selected covariates as potentially confounding pretreatment variables to minimize biased estimates.

[1] Hernán, M. A., & Robins, J. M. (2016). Using Big Data to Emulate a Target Trial When a Randomized Trial Is Not Available. American Journal of Epidemiology, 183(8), 758-764. <https://doi.org/10.1093/aje/kwv254>

[2] Hernán, M. A., Wang, W., & Leaf, D. E. (2022). Target Trial Emulation: A Framework for Causal Inference From Observational Data. JAMA, 328(24), 2446-2447. <https://doi.org/10.1001/jama.2022.21383>

1.3. Considering you propose the use of the average marginal effects, I really miss the reporting of a causal framework (AME is used as a proxy of the ATE). I cannot understand why you did not consider the usefulness of drawing your assumptions before making conclusions (and stay only as an association having the data and the scientific ambition). May you consider it useful to draw a DAG regarding this potential causal relationship -if you condeir this-. Please consider adding a DAG to directly address 1) a causal pathway, 2) a mediator, 3) potential confounders, and/or 4) colliders in the causal structure. Do you consider if some of the included adjusted variables in your models could be a collider and not a confounder? If you are only considering association, well, that is fine but should clarify and improve your conclusions.

> Since we used logistic regression models to analyze our dichotomous outcomes, we decided to report the Average Marginal Effects (AME) in addition to the adjusted odds ratios to facilitate understanding and interpretation of the results. Regression coefficients in generalized linear models (GLM), which quantify conditional stochastic dependencies between a predictor and a limited or categorical dependent variable, can be quite unintuitive. Additionally, comparing regression coefficients across models with different predictors or covariates is limited due to the issue of non-collapsibility [1, 2]. To present our findings in a clear and accessible manner, we have chosen to use AMEs. These represent the covariate-adjusted risk differences in the outcome when a specific predictor changes by one unit, while keeping other predictors in the model constant [3, 4]. For dichotomous predictors or appropriately dummy-coded categorical predictors, the AME indicates the covariate-adjusted risk difference between the groups.

We acknowledge that the term "average marginal effect" can be potentially misleading, as the AME is no more or less "causal" than the regression coefficient (i.e., the adjusted log odds ratio). As outlined above, the causal inference relies on the validity of key assumptions rather than on the choice of metrics used to present the results of a statistical analysis. However, we have decided not to use alternative terminology in order to remain compatible with the existing literature.

We agree with the reviewer that the AME derived from a binary logistic regression using observational data is an unbiased estimator of the ATE in a target population if (a) the assumption of strong ignorability holds, (b) the covariates are measured without bias (perfect reliability), (c) the model is correctly specified, and (d) the sample is representative of the target population. So we have included the following paragraph in the discussion:

*“We suspect that adequate pain control after major surgery has an influence on the risk of postoperative complications. However, drawing causal conclusions from observational studies relies on certain assumptions, as outlined in the Neyman-Rubin causal model (NRCM). The Average Marginal Effect (AME), derived from the logistic regression model, serves as an unbiased estimator of the Average Treatment Effect (ATE) under the conditions that (a) all confounding variables are properly accounted for (i.e., strong ignorability and correct model specification) and that these covariates are accurately measured. However, due to the study's design, we cannot completely eliminate the possibility of unmeasured confounders, which can introduce residual bias in the adjusted odds ratio (OR) and AME”.*

We feel that the suggestion to present a DAG seems not directly related to the reporting AMEs. In fact, DAGs are a useful tool for visualizing assumed causal and non-causal (e.g., implicit) relationships between random variables and identifying groups of potentially confounding variables.

However, in our study we finally decided not to present DAGs as we did not analyse causal processes over time, with several intermediate variables (e.g., mediators).

The focus of our study is to evaluate the appropriateness of pain control in comparison to inappropriate pain control. This is analogous to the treatment variable (i.e., exposure). As discribed in the section titled “Patient characteristics and covariates”, we have intentionally selected potential confounding variables for each outcome. We took great care to include only covariates that could not themselves be influenced by the treatment variable. In other words, all post-treatment or post-exposure variables were precluded to avoid an endogenous selection bias / collider bias [5, 6].

No effect moderating variables or mediators were considered. The (non)causal relationships among the set of selected covariates (i.e., potential confounders) are not relevant for the estimation of the stochastic association between the exposure variable and the outcome. Accordingly, the DAGs that account for the temporal order of the selected variables in our analyses is quite simple (covariates => exposure, covariates => outcome, exposure => outcome) and structurally equivalent for all considered outcomes. Therefore, we refrained from presenting the DAG.

However, we explicitly note in the revised manuscript that the temporal order was taken into account in the selection of covariates. In the section “Patients and covariates” we now state: *“Only those covariates were considered as potential confounders that were temporally prior to the exposure to avoid an endogenous selection bias (i.e., collider bias).”*

[1] Daniel, R., Zhang, J., & Farewell, D. (2021). Making apples from oranges: Comparing noncollapsible effect estimators and their standard errors after adjustment for different covariate sets. Biometrical Journal, 63(3), 528-557. <https://doi.org/https://doi.org/10.1002/bimj.201900297>

[2] Sjölander, A., Dahlqwist, E., & Zetterqvist, J. (2016). A Note on the Noncollapsibility of Rate Differences and Rate Ratios. Epidemiology, 27(3), 356-359. <https://doi.org/10.1097/ede.0000000000000433>

[3] Onukwugha, E., Bergtold, J., & Jain, R. (2015). A primer on marginal effects—Part I: Theory and formulae. Pharmacoeconomics, 33(1), 25-30. https://doi.org/10.1007/s40273-014-0210-6

[4] Onukwugha, E., Bergtold, J., & Jain, R. (2015). A primer on marginal effects—part II: health services research applications. Pharmacoeconomics, 33(2), 97-103. <https://doi.org/10.1007/s40273-014-0224-0>

[5] Elwert, F., & Winship, C. (2014). Endogenous Selection Bias: The Problem of Conditioning on a Collider Variable. Annual Review of Sociology, 40(Volume 40, 2014), 31-53. <https://doi.org/https://doi.org/10.1146/annurev-soc-071913-043455>

[6] Montgomery, J. M., Nyhan, B., & Torres, M. (2018). How Conditioning on Posttreatment Variables Can Ruin Your Experiment and What to Do about It. American Journal of Political Science, 62(3), 760-775. https://doi.org/https://doi.org/10.1111/ajps.12357

1.4. The reported complications, (lines 260-261 “In patients with inadequate pain control, at least one postoperative complication occurred more frequently (22%) than in the adequately treated group (16%).”) should be reported besides their CI95% bc those are direct estimates of risk.

> Thank you for bringing this to our attention; however, there seems to be a misunderstanding. The percentages mentioned do not reflect direct risk estimates. Instead, they represent relative frequencies within the context of a descriptive analysis of postoperative complications. We have revised the sentence for improved clarity: “In our study cohort, patients with inadequate pain control were more likely to experience at least one postoperative complication (in 21% of cases) than those with adequate pain control (12%, P = 0.024).”

1.5. The definition of “prolonged analgesic use” as a proxy for chronic pain is reasonable but could benefit from validation or at least a more detailed justification, given the lack of a diagnostic ICD code.

> Thank you for this comment. In 2021, Elisabeth Nilsen et al. established a definition for prolonged postoperative opioid use (PPOU) [1], which categorizes opioid use exceeding 90 days after surgery as prolonged. We applied the same definition for prolonged postoperative analgesic use, which includes opioids, non-opioids, and/or co-analgesics. A similar definition has been used by Yu et al. [2]. Since we received health insurance data on a quarterly basis, we needed to make some adjustments. We defined prolonged postoperative analgesic use as having at least one prescription for an opioid (or non-opioid or co-analgesic) during the first 90 days after discharge, along with at least one additional prescription for these drug classes between days 91 and 180. This approach ensured that the medications were used for more than 90 days following the surgical procedure. We have specified this in the “Outcomes” section.

[1] Nilsen EA, Mahmoud KF, Mitchell AM, Germack HD. Creating an operational definition for prolonged postoperative opioid use through the use of concept analysis. Appl Nurs Res. 2021 Dec;62:151506. doi: 10.1016/j.apnr.2021.151506. Epub 2021 Oct 6. PMID: 34815002.

[2] Yu CH, Chen YC, Hung IY, Chen JY, Chang YJ, Ho CH, Chu CC. Long-term analgesic and opioid prescription after surgery under general or neuraxial anesthesia: A retrospective nationwide sampling study. J Clin Anesth. 2021 Dec;75:110438. doi:10.1016/j.jclinane.2021.110438. Epub 2021 Jul 24. PMID: 34311243.

1.6. The exclusion of patients with incomplete pain data could introduce bias. Authors should provide a sensitivity analysis or at least a discussion of this potential issue. There is no major information about the management of missing data.

> Thank you for these two comments, with which we agree. The first relates to a possible bias caused by incomplete pain data. Our study included patients who were scheduled to stay in hospital for at least three days after surgery. However, due to the special circumstances surrounding the coronavirus pandemic, patients were discharged earlier in rare cases and were then no longer available for questioning (usually on day 3). The definition of adequate pain control was deliberately chosen to be very strict and was only considered to have been met if the NRS scores collected on all 3 days were <4. Consequently, patients who did not meet this criterion and therefore could not be reliably assigned to the group with adequate pain control were excluded from the analysis (listwise deletion). A total of 16 patients (3.0%) had to be excluded from the analysis of (in)adequate pain control. Twenty-seven patients (5%) had to be excluded from the analysis of pain peaks. According to Enders et al. [1], these rates of missing data are considered irrelevant.

The second comment refers to missing information on how to deal with missing data. We apologize for the incomplete information and specified our approach in the “Exposures” section. Statistical missing data theory implies that statistical inference with incomplete data is unbiased under certain conditions/assumptions. Listwise deletion leads to decreased efficiency and power in parameter estimation but yield unbiased parameter estimates if the missing data mechanism is missing completely at random. This is an assumption which may not hold true in our study.

We added therefore a statement in the “Discussion” section:

“*We used listwise deletion for patients with incomplete records (e.g., incomplete pain scores over time). Unbiased parameter estimation using listwise deletion requires that the missing data mechanism is missing completely at random, which cannot be ensured in our study. However, since no more than five percent of cases were affected in any analysis, no relevant bias can be assumed.*”

[1] Enders, C.K. (2010). Applied Missing Data Analysis. Guilford Press.

[2] Rubin, D. B. (1976). Inference and missing data. Biometrika, 63. <https://doi.org/10.1093/biomet/63.3.581>

[3] Little, R. J., & Rubin, D. B. (2019). Statistical analysis with missing data (3 ed.). John Wiley & Sons.

1.7. Pain scores are patient-reported but also dependent on measurement frequency and method, which may vary between institutions. Addressing potential inter-center variability in pain assessment could influence those reporting. Please may you discuss something about.

> Thank you for this comment. We are well aware of the problem of possible inter-center variability and have discussed this aspect in the statistical model creation process. Since this trial was conducted prospectively, we were able to provide instructions to the participating centers on how to collect data. This ensured that the data processed for this analysis was as complete and uniformly accurate as possible. Data collected aligned with the dataset specified by the net-ra project for regular therapy documentation where the pain assessment method is standardized. Intermittend checks of data quality with the participating centers did not reveal any deviation. Therefore, we believe that the inter-center effect is not relevant in this analysis. We clarified this in the Methods section.

1.8. Discussion does not mention at all any intent to interpret the result as causal. There are four (or five) major assumptions of causality in cohort studies and big data. Exchangeability, Positivity, Consistency, Temporality and Target population. Please address each one in your discussion in order to assess if your study fits some of them (if any) and their interpretation with a causal approach.

> We addressed the issue of exchangebility explicitely in the limitation section (lines 480-86).

The positivity assumption is reasonable as all included hospitalized patients had the a priori chance of an adequate pain treatment after a surgery.

The consistency assumption requires a clear definition of the treatment or exposure to ensure unambiguous potential outcomes for all levels of the treatment/exposure variable. In our study, we used three different operationalizations, with clear and objective criteria to define the exposure levels. Therefore, consistency can be assumed for each operationalization.

As previously explained, we considered the temporal sequence of the variables and events in our study. First, we focused only on covariates as potential confounders that existed prior to “treatment” to avoid endogenous selection or collider bias. Second, all results refer to events or complications that occurred after the observation period for (in)adequate pain control. Please note that we did not investigate the "directness" of potential effects of pain therapy or the causal pathways through intermediate variables after the postoperative pain treatment period in the hospital. From the perspective of the NRCM, this does not limit our ability to estimate the treatment effect in relation to the results. It simply indicates that the causal mechanisms underlying the presumed effect have not been empirically explored. However, investigating these mechanisms was not the focus of our study and could not be achieved with the available data.

The target population consists of hospitalized patients with surgical treatments who are eligible for postoperative pain treatment. Representativeness of the sample regarding the target population and therefore generalizability of the results are potentially limited due to recruitment of patients based on the net-ra register and the BARMER health ensurance. We explicitly state this limitation in the manuscript.

We had an in-depth discussion about how to respond to this reviewer's comment. While it is important to address the underlying theory of causality and its assumptions, we must also consider that our paper is not a methodological article; rather, it is an applied research study. Therefore, we believe the discussion should center on the study itself, including its strengths and weaknesses, rather than delving into the statistical theory of causal inference. To maintain the focus on the impact assessment of postoperative pain control, we have decided to reference the relevant assumptions from the literature while only discussing those that are crucial to our study. We have included the following paragraph in the "Discussion" section:

*“We suspect that adequate pain control after major surgery has an influence on the risk of postoperative complications. However, drawing causal conclusions from observational studies relies on certain assumptions, as outlined in the Neyman-Rubin causal model (NRCM) [26, 27]. The Average Marginal Effect (AME), derived from the logistic regression model, serves as an unbiased estimator of the Average Treatment Effect (ATE) under the conditions that (a) all confounding variables are properly accounted for (i.e., strong ignorability and correct model specification) and that these covariates are accurately measured. However, due to the study's design, we cannot completely eliminate the possibility of unmeasured confounders, which can introduce residual bias in the adjusted odds ratio (OR) and AME. Generalizability of the results is potentially limited as the representativeness of the sample with respect of the target population is not ensured. This is due to the recruitment of only patients from the net-ra register who are insured with BARMER health insurance.”*

Minor comments

Abstract

1.9. The study addresses an important and underexplored association between early postoperative pain control and surgical outcomes. I may say the abstract is not well written and perhaps incomplete. Methods section of the abstract should be improved to improve the overall understanding of the process. Add exact sample size (n=539) to enhance transparency.

> Thank you for pointing this out. Fortunately, PLOS ONE allows a higher word count than usual for the abstract. This made it possible to add further information.

Introduction:

2.1. First two lines (69-72) require a reference and I consider there are plenty of.

> Thank you for pointing this out. We added the references to the relevant sentences.

2.2. Lines 73-74 states you are expanding about association of. Is that the cause or not? Is there any studies focused on studying -using proper analytical methods- causality in this scenario of much of the literature is discussion association.

2.3. Line 76-77 suggests a potential cause of the complications beyond any association.

2.4. Lines 78-85. Is that enough for association studies? Why not address the problem of causality more focused on. Or please expand about the analytical nature of those mentioned studies.
I strongly miss the main objective of this study. I mean, both hypotheses sound interesting but please state clearly the aims of this study including if your aiming causation or only association. From the Record declaration you mentioned later on “Because the strengths and limitations of methods used to conduct research with routinely collected data may be contentious, a clear description of a study’s objectives is essential”.

> We feel that these comments (2.2.-2.4.) address the main issue of causality raised by the reviewer in his major comments. We hope that our responses above, as well as the revisions in our manuscript, are sufficient to address the concerns regarding causality vs. association.

We have revised the lines mentioned to clearly establish the basis for our analysis, and we have re-evaluated the literature referenced in those lines concerning the concept of causality and the methodologies employed. Indeed, the described associations found in the literature are hypothesized (“*possible consequences*”) to be causal in this sentence. Our hypothesis that an adequate pain control affects the risk of postoperative complications is based on this idea.

Methodology:

2.5. Prospective design with large sample size and linkage to real-world insurance data adds robustness. Please state in line 100 you are using routinely collected data.

> Thanks for pointing that out. We have added to the sentence in question that we used routinely collected data.

2.6. I can see you selected high risk patients for postop severe pain (lines 134-136 “Only patients who were insured with BARMER GEK, for whom a complex pain therapy procedure and a minimum length of stay of three days were planned, were asked to participate in the study and provide informed consent.”

> Yes, that's correct. As we were able to show in a retrospective secondary data analysis of 203,327 patients using health claims data, minor surgical procedures are not relevant in terms of prolonged postoperative opioid use [1].

[1] Dreiling J, Rose N, Arnold C, Baumbach P, Fleischmann-Struzek C, Kubulus C, Komann M, Marschall U, Rittner HL, Volk T, Meißner W, Schwarzkopf D: The incidence and risk factors of persistent opioid use after surgery – a retrospective secondary data analysis. Dtsch Arztebl Int 2024; 121. DOI: 10.3238/arztebl.m2024.0200

2.7. Please clarify lines 177-179 “To ensure that the complications mentioned were not the reason for hospitalization but occurred after the surgical procedure, we only considered secondary diagnoses and not principal diagnoses.”

> Unfortunately, health claims data in Germany only records ICD codes for diagnoses during a hospital stay without time stamps. We therefore had to define how to deal with this. Primary diagnoses are typically the conditions that lead to the hospital stay. In our cohort, these were diagnoses associated with the surgical procedure performed. Diagnoses that arise during the inpatient stay are typically classified as secondary diagnoses. We therefore decided to use only secondary diagnoses for the complications examined. We have clarified the sentence in the manuscript.

2.8. The exclusion of patients with incomplete pain data could introduce bias. Authors should provide a sensitivity analysis or at least a discussion of this potential issue. There is no major information about the management of missing data.

> This comment appears twice – we have answered it in detail under point 1.6.

Results

2.9. Figure 2 is informative but dense. Consider separating pain outcomes and complication outcomes for clarity.

> We agree and have separated the graphical representation of the results for both outcomes (formerly Figure 2, now Figures 2 and 3).

3.0. Please use consistent terminology throughout (e.g., "chronic postsurgical pain" vs. “prolonged analgesic use”).

> Thanks for pointing that out. We have revised the manuscript to ensure the correct use of these two terms.

3.1. The main adjusted regression model for the primary outcome under study should be presented in the main text being carefully to avoid any interpretation of other coefficients (which in general is correct and appropriate).

> Thank you for your comment. As you correctly noted, the interpretation of effect estimators for the covariates is limited. Therefore, we have intentionally reported only the results for the treatment variables in the main text and have additionally listed the covariates used in our model adjustments. You can find the complete results tables for all models in the supplementary material (S7 Table). To prevent misinterpretation, we would like to keep the focus on our main results.

Discussion

3.2. First paragraph of the discussion is very clear.
Second paragraph is more useful. I would suggest making a Table regarding the availability of similar studies or studies addressing the same association.

> To the best of our knowledge, the studies referenced in this paragraph are the only studies available on the subject of postoperative acute pain and complications. Given the limited number of studies available, we would prefer to stick to the text form.

3.3. Final conclusion, however includes a causal statement that should be considered “Postoperative acute pain should, therefore, be consistently assessed until
resolvement, and all treating specialists should strive to minimize pain load.”

> Thank you for this comment. We have revised the manuscript regarding the concept of causality, and our results do not contradict a causal relationship. We, therefore, would like to stand by this cautious causal interpretation.

We thank the reviewer once again for encouraging us to pay closer attention to methodology, both now and especially in future observational studies, to be able to examine causal relationships.

Reviewer #2

Review PONE-D-25-25651
Quality of pain release after major surgery and its association with postoperative complications – a prospective observational study

In general:
Thank you for the invitation to review this paper.
In this prospective multicenter observational study the authors tested the hypothesis that postoperative complications occur more frequently in patients with poor pain control regarding both severity and duration. Second, that the likelihood of pain chronification is higher in these patients, reflected by prolonged postoperative analgesic consumption.
The study is well-designed and makes original use of various linked registries. Considering the individual time that a patient needs to overcome postoperative pain, which means having stable, at most mild pain levels, as a new approach is interesting.
In the use of the registries, understandable methodological choices have been made, which may, however, influence the results. I therefore suggest examining these choices carefully and providing a more detailed discussion of potential limitations and points for consideration.

> Thank you for the positive assessment. We have expanded the discussion accordingly, particularly to include the concerns listed below.

Major comments:

Methods:

1.1. The inclusion of only patients insured with BARMER GEK may introduce a risk of selection bias, as membership in this statutory health insurance fund may not be representative of the general German population. Individuals insured by BARMER may differ systematically from those covered by other statutory or private insurers with respect to socioeconomic status, age, health status, or regional distribution. For example, higher-income individuals are more likely to opt for private health insurance, and BARMER may have a higher proportion of older or chronically ill patients compared to smaller or more specialized funds. Therefore, the study findings may have limited generalizability beyond the insured population of BARMER GEK and introducing other risk factors for postoperative pain. I suggest to comment this in the discussion section.

> Thank you for this comment, with which we agree. BARMER health data is regularly used for scientific analysis [1-5]. As we explained in the section on study design, BARMER (BARMER GEK is an outdated term, which we have corrected in the manuscript) is the second-largest statutory health insurance fund in Germany and provides coverage for around 9 million insured persons. It is open to all persons who are subject to compulsory statutory insurance, as is the case with all other statutory health insurance funds (e.g., Techniker, DAK, AOK). Therefore, it can generally be assumed that the demographic structure of the population insured by BARMER is representative of the population with statutory health insurance, which accounts for approximately 90% of the total German population. According to BARMER, compared to other statutory health insurance companies, its insured persons are on average more likely to be female and younger. We have taken both factors into account in our statistical models. During the survey period, the number of privately insured individuals who were not included in our analysis totaled approximately 8.7 million, which corresponds to about 10% of the German population [6,7]. Privately insured individuals differ from those with statutory insurance in various aspects. To minimize bias related to differences in health status, we accounted for key factors such as sex, age, BMI, and the American Society of Anesthesiologists' Physical Status Scorein our statistical models. Since residual bias cannot be ruled out and generalizability is limited as a result, we have included this in the “Discussion” section.

[1] Peters F, Kuchenbecker J, Acar L, Marschall U, L'Hoest H, Lareyre F, Spanos K, Behrendt CA. Antithrombotic Treatment Patterns of Patients with Symptomatic Peripheral Arterial Occlusive Disease in Germany: Evidence from Health Insurance Claims Data. J Clin Med. 2022 Sep 16;11(18):5455. doi: 10.3390/jcm11185455. PMID: 36143102; PMCID: PMC9501602.

[2] Köhler, F., Acar, L., van den Berg, A. *et al.* Correction to: Impact of the COVID-19 pandemic on appendicitis treatment in Germany—a population-based analysis. *Langenbecks Arch Surg* **407**, 3917 (2022). <https://doi.org/10.1007/s00423-021-02343-1>

[3] Eva Freisinger, Joachim Gerß, Lena Makowski, Ursula Marschall, Holger Reinecke, Helmut Baumgartner, Jeanette Koeppe, Gerhard-Paul Diller, Current use and safety of novel oral anticoagulants in adults with congenital heart disease: results of a nationwide analysis including more than 44 000 patients, *European Heart Journal*, Volume 41, Issue 43, 14 November 2020, Pages 4168–4177. <https://doi.org/10.1093/eurheartj/ehaa844>

[4] Kaschwich, M., Peters, F., Hischke, S., Rieß, H. C., Gansel, M., Marschall, U., L’hoest, H., Heidemann, F., Debus, E. S., Acar, L., Kreutzburg, T. & Behrendt, C.-A. (2020). Long-term incidence of cancer after index treatment for symptomatic peripheral arterial disease – a health insurance claims data analysis. VASA. Zeitschrift Fur Gefasskrankheiten, 49(6), S. 493–499. <https://doi.org/10.1024/0301-1526/a000901>

[5] Acar L, Peters F, Marschall U, L'Hoest H, Twine C, Behrendt CA. Increased Pulmonary Embolism Incidence and Mortality in Patients Subsequently Diagnosed with COVID-19: An Analysis of Health Insurance Claims Data. Eur J Vasc Endovasc Surg. 2022 Jan;63(1):159-160. doi: 10.1016/j.ejvs.2021.08.027. Epub 2021 Sep 6. PMID: 34627680; PMCID: PMC8418913.

[6] https://de.statista.com/statistik/daten/studie/155823/umfrage/gkv-pkv-mitglieder-und-versichertenzahl-im-vergleich/

[7] https://de.statista.com/statistik/daten/studie/2861/umfrage/entwicklung-der-gesamtbevoelkerung-deutschlands/

1.2. When considering patients to have inadequately controlled postoperative pain if the NRS scores with movement were above three at least once on days 1-3 after surgery (according to the cut-off for moderate to severe pain described by Gerbershagen), this means that patients with an NRS4 once in those three days are considered to have inadequate pain control. Although used in other studies, to my clinical opinion this is far to strict and may have lead to more patients in the inadequate pain control group.
When checking literature, the results of the paper of van Boekel et al.(2017) reinforces my clinical opinion. I would suggest not only stating that the cut-off is widely used, but also discussing the cut-off in the light of personalized levels of pain scores.

*van Boekel, R. L. M., Vissers, K. C. P., van der Sande, R., Bronkhorst, E., Lerou, J. G. C., & Steegers, M. A. H. (2017). Moving beyond pain scores: Multidimensional pain assessment is essential for adequate pain management after surgery. PloS one, 12(5), e0177345.* [*https://doi.org/10.1371/journal.pone.0177345*](https://doi.org/10.1371/journal.pone.0177345)

> We certainly agree that this definition is very strict and we are very well aware of the advantages of a multidimensional pain assessment in a holistic biopsychosocial pain model. However, the basis for using this model in the acute postoperative situtation is less well established and subject of current debates [1]. Furthermore, it is not yet part of the routine documentation of acute pain services. We agree that the term “widely used” has little substance. The chosen cut-off between none to mild (NRS 0-3) and moderate to severe postoperative pain (4-10) was also used by van Boekel et al. in 2019 to distinguish irrelevant from relevant pain [2]. In this trial, pain was also considered unacceptable if it was rated as unacceptable by the patient only once within the first three postoperative days. Our goal was to clearly separate patients who reported no relevant pain from those who reported relevant pain in the form of prolonged pain of more than 4 on the NRS scale or in the form of (at least one) pain peak. We are aware that there can be many gradations of “pain burden” within the range of relevant pain, which in turn can influence the postoperative course. Further research focusing on this specific topic is needed. We have expanded the relevant section in the discussion to provide more clarity.

[1] Pogatzki-Zahn EM, De Lucia S, Weinmann C, Heitkamp H, Hummelshoj L, Liedgens H, Meissner W, Vincent K, Vollert J, Zahn P; IMI-PainCare PROMPT Consensus Panel; Kaiser U, Rosenberger DC. A core outcome set of measurement instruments for assessing effectiveness and efficacy of perioperative pain management: results of the international IMI-PainCare PROMPT Delphi consensus process. Br J Anaesth. 2025 May;134(5):1460-1473. doi: 10.1016/j.bja.2025.01.029. Epub 2025 Mar 14. PMID: 40089403; PMCID: PMC12106875.

[2] van Boekel RLM, Warlé MC, Nielen RGC, Vissers KCP, van der Sande R, Bronkhorst EM, Lerou JGC, Steegers MAH. Relationship Between Postoperative Pain and Overall 30-Day Complications in a Broad Surgical Population: An Observational Study. Ann Surg. 2019 May;269(5):856-865. doi: 10.1097/SLA.0000000000002583. PMID: 29135493.

Results:

1.3. Mentioned in text of results (page 15, line 265): “In patients with inadequate pain control, at least one postoperative complication occurred more frequently (22%) than in the adequately treated group (16%).” (p-value missing). However, in table 2 it shows that “Composite: at least one of the above complications” is 21% versus 12%? Please explain.

> Thank you for pointing out this error. The figures in the text were incorrect and have now been corrected. We have added the missing p-value at this point and now consistently report all p-values in the results.

1.4. Mentioned in text of results (page 18, line 316): “Pain peaks during the first three postoperative days and the speed of pain recovery had no influence on the occurrence of chronic postoperative pain in our analysis (pain peaks: 0.02; 95% CI -0.05 to 0.10; P=0.555; pain recovery: -0.01; 95% 319 CI -0.07 to 0.06; P=0.836).” I think you mean “prolonged postoperative analgesic use“ as a proxy for chronic postoperative pain, as stated in methods? Please explain.

> Thank you for pointing this out. We apologize for this inaccuracy and have corrected the sentence accordingly: “Pain peaks during the first three postoperative days and the speed of pain recovery had no influence on the occurrence of prolonged postoperative analgesic use in our analysis …”

Discussion:

1.5. You were unable to demonstrate any influence of the aforementioned pain therapy characteristics on prolonged postoperative analgesic use. I would suggest to add a paragraph in the discussion to discuss prolonged postoperative analgesic use as a proxy for chronic postoperative pain, in the light oft he German culture of opioid prescribing which may differ from other countries?

> The long-term use of analgesics is particularly important when opioids are involved. We estimated the overall rate of long-term postoperative opioid use in Germany to be 1.4% [95% CI 1.4–1.5%] based on a sample of 203,327 opioid-naive patients who underwent surgery [1]. This rate appears to be significantly lower than in other countries. Recently, the rule that patients may not be prescribed opioids upon discharge has been replaced by a special prescription form that specifies the lowest possible amount and a validity of only 3 days. This is similar to Florida's HB 21 law from 2018, but is more conservative than the regulations in Michigan, where a 14-day prescription of opioids is permitted.

Among non-opioids, aspirin, diclofenac, ibuprofen, naproxen, and acetaminophen are available in Germany without a prescription in low doses and limited quantities. This contrasts sharply with some non-opioid analgesics in the US, where thousands of tablets per bottle are freely available.

Since the amount of freely available non-opioids is difficult to control, we only use prescription non-opioids in our analysis to be more robust.

[1] Dreiling J, Rose N, Arnold C, Baumbach P, Fleischmann-Struzek C, Kubulus C, Komann M, Marschall U, Rittner HL, Volk T, Meißner W, Schwarzkopf D: The incidence and risk factors of persistent opioid use after surgery – a retrospective secondary data analysis. Dtsch Arztebl Int 2024; 121. DOI: 10.3238/arztebl.m2024.0200

Minor:

1.6. Abstract: Please correct objektives in objectives

> Thank you for noticing this. We have corrected the typo.

1.7. Please add % to numbers of patients in results, for example page 16, line 270. And p-values when comparing results, for example page 15, line 261.

> Thank you very much for this comment. We have added the missing percentages to the numbers in the results section. As already explained in point 1.3, we have also added the missing P-values.

1.8. In results is stated: “were more than twice as high for patients with inadequately controlled pain (adjOR 2.56; 95% CI 1.43 266 to 4.80, P=0.002).” The same results are described in the abstract as: “had almost three times the risk of complications (adjOR 2.56; 48 95% CI 1.43-4.80, p=0.002)”. In my opinion, 2.56 is not almost 3, please adjust.

> Thanks for pointing that out. We have corrected the relevant sentence in the abstract.

1.9. Table 1, what do you mean with “Definition not applicable” underneath table 1?

> Thank you for this comment. We agree that this is misleading. Our study included patients who were supposed to stay in the hospital for at least three days after surgery. However, due to the special circumstances surrounding the coronavirus pandemic, patients were discharged earlier in rare cases and were then no longer available for interviews (usually on the third day). The definition for the absence of pain peaks (as well as for adequate pain control) was deliberately chosen to be very strict and was only considered to be met if the NRS values collected on all three days were <7. If the NRS values collected were <7 and the value for at least one day was missing, patients could not be assigned to the “no pain peaks” group and were therefore excluded from the analysis. We have deleted the misleading wording and now report the patients excluded due to incomplete pain data both in the manuscript text (Results section) and in the table legend.

_____________________________________________________________________
